# A plug-and-play aptamer diagnostic platform based on linear dichroism spectroscopy

**DOI:** 10.3389/fchem.2023.1040873

**Published:** 2023-05-09

**Authors:** Haydn A. Little, Aysha Ali, Jake G. Carter, Matthew R. Hicks, Timothy R. Dafforn, James H. R. Tucker

**Affiliations:** ^1^ School of Chemistry, University of Birmingham, Birmingham, United Kingdom; ^2^ School of Biosciences, University of Birmingham, Birmingham, United Kingdom; ^3^ Linear Diagnostics Ltd., Birmingham, United Kingdom

**Keywords:** aptamer, diagnostics, DNA, bionanoparticle, M13, sandwich assay

## Abstract

A plug-and-play sandwich assay platform for the aptamer-based detection of molecular targets using linear dichroism (LD) spectroscopy as a read-out method has been demonstrated. A 21-mer DNA strand comprising the plug-and-play linker was bioconjugated onto the backbone of the filamentous bacteriophage M13, which gives a strong LD signal due to its ready alignment in linear flow. Extended DNA strands containing aptamer sequences that bind the protein thrombin, TBA and HD22, were then bound to the plug-and-play linker strand via complementary base pairing to generate aptamer-functionalised M13 bacteriophages. The secondary structure of the extended aptameric sequences required to bind to thrombin was checked using circular dichroism spectroscopy, with the binding confirmed using fluorescence anisotropy measurements. LD studies revealed that this sandwich sensor design is very effective at detecting thrombin down to pM levels, indicating the potential of this plug-and-play assay system as a new label-free homogenous detection system based on aptamer recognition.

## 1 Introduction

The SARS-CoV-2 pandemic has starkly highlighted how molecular diagnostics can make a very significant impact on healthcare outcomes. It is clear that both the antibody-based lateral flow device (LFD) test and DNA-based tests like PCR have saved countless lives and reduced the global severity of the pandemic. But nevertheless the diagnostic response to the outbreak has also shown that there is still much room for improvement in modern healthcare diagnostics. (Alafeef and Pan, 2022). Even established tests that use PCR and LFDs can suffer from a combination of various issues such as time and sensitivity, as well as limitations in throughput. In addition, nucleic acid tests such as PCR are largely restricted to the laboratory, slowing down the testing process further and in some instances negating their use. These observations make it clear that efforts need to continue in generating new testing modalities to resolve these issues. This paper details the next stage of development of our bacteriophage/linear dichroism (LD) assay system that offers an alternative target read-out method to more established techniques. (Michel et al., 2020; Trotter et al., 2020; Sun et al., 2019; Chen et al., 2019). This system has been previously developed for detection using antibodies (Pacheco-Gómez et al., 2012) and DNA (Ali et al., 2020) and offers high levels of sensitivity alongside speed and simplicity. Here we extend our work on this signal read-out methodology to encompass DNA aptamer-based detection.

The LD/bacteriophage assay is based on the observation that filamentous bacteriophage (equivalent to a filament that is 1 μm long by 10 nm wide) can be aligned in fluid flow. This in turn aligns the chromophores within the bacteriophage, which can then be detected using LD spectroscopy. Crucially any other material present in solution that does not align has no LD signal, meaning that impurities do not perturb the spectrum. In previous work, we exploited this observation for an assay by derivatising a bacteriophage (M13 in this case) with moieties that could bind to an assay target at physiological pH. Upon target binding, the hydrodynamic behaviour of the particle changed, altering alignment and hence the LD signal. We accordingly demonstrated that this novel LD sensing methodology could be used to detect pathogenic *E. coli* using antibodies (Pacheco-Gómez et al., 2012) as well as DNA from pathogens. (Ali et al., 2020).

**SCHEME 1:**
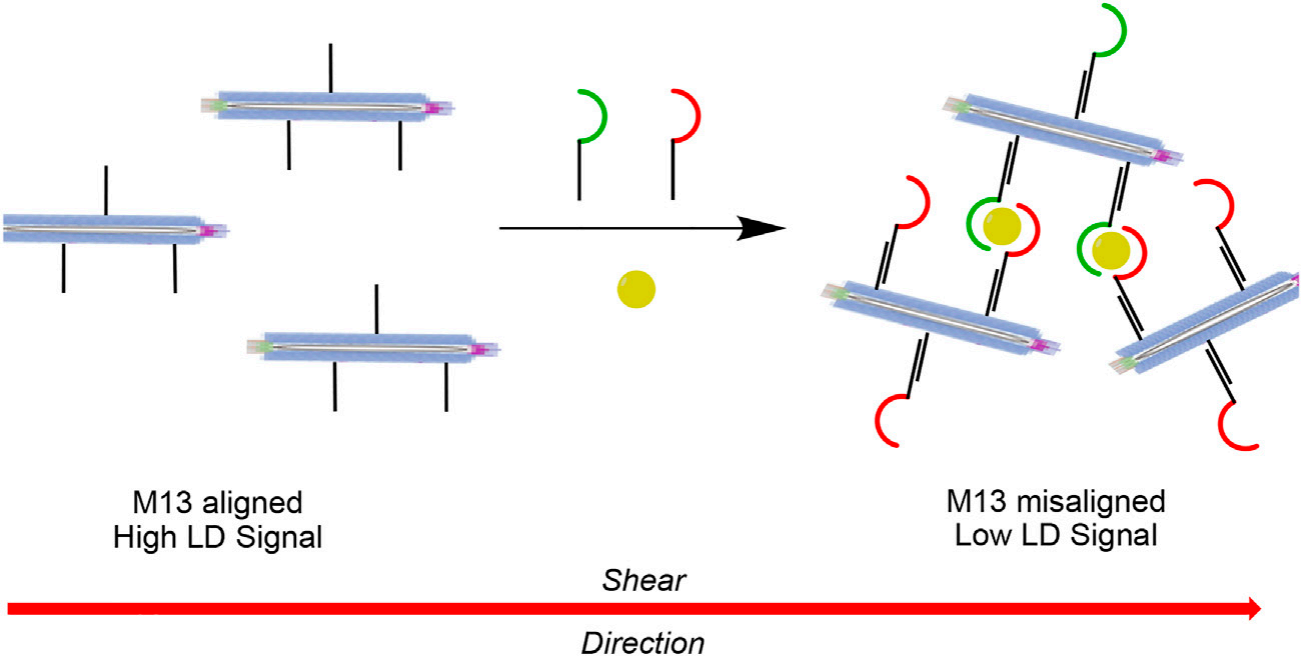
Schematic representation of the plug-and-play method for target recognition using LD spectroscopy. Each M13 bacteriophage is covalently attached to copies of the same DNA strand, which are then bound to complementary DNA strands containing one-half of a recognition element for the target of interest. The presence of the target (shown in yellow) causes aggregation of the particles in solution through the formation of multiple sandwich complexes, which results in misalignment of the particles in shear flow and hence a reduction in the LD signal.

In the case of DNA sensing using LD spectroscopy, we generated a “sandwich assay” where the DNA target bridges between two M13 bacteriophages in order to disrupt alignment. (Ali et al., 2020). We found it sufficient to covalently attach DNA strands to the **M13** bionanoparticles that were complementary to different sequence regions of the target to bring about a change in LD signal. However, in the case of detecting a non-nucleic acid target using similar DNA-functionalised particles, a slightly more intricate design was required. Here we show the proof-of-concept of a so-called plug-and-play method for target recognition using LD spectroscopy (Scheme 1). It consists of a highly modular **M13** sensing platform that in principle can be used for interaction with any combination of aptameric sequence for which target binding brings about a change in LD signal.

## 2 Results and discussion

### 2.1 Design

In order to demonstrate the desired change in **M13** alignment for LD sensing of non-DNA targets using aptamers, it was decided to continue to explore our sandwich assay design that had already been shown to be highly effective for DNA sensing. (Ali et al., 2020). For the target, we chose the protein thrombin, the main regulatory enzyme in the blood coagulation cascade, for two reasons. Firstly its aptamer binding properties are well characterized and understood, making it a popular and highly effective target exemplar for a number of other sensor platforms. (Ikebukuro et al., 2004; Xiao et al., 2005; Wang et al., 2008; Cho et al., 2008; Wang and Zhao, 2012; Deng et al., 2014; Kim et al., 2018; Malecka and Ferapontova, 2021; Neupane and Stine, 2021). Secondly two different thrombin aptamers are known (**TBA** and **HD22**), enabling a relatively straightforward design for a sandwich assay, which involves the simultaneous binding of different regions of the thrombin by two separate DNA strands. Indeed others have already demonstrated the effectiveness of this approach in various sandwich assays for thrombin (Neupane and Stine, 2021; Ikebukuro et al., 2004; Wang and Zhao, 2012).

As we had previously demonstrated an effective bioconjugation technique involving thiol-ene and amide coupling chemistries to append DNA strands to lysine residues (pVIII protein) of the **M13** bacteriophage, (Carr-Smith et al., 2015; Ali et al., 2020), we decided to adopt the same approach here. However instead of directly attaching the two different aptamers to the **M13** chassis, we used exactly the same linker strand as the one employed previously for DNA sensing (Ali et al., 2020). Using this approach, the same functionalised chassis can be connected via DNA base-pairing to any aptamer of choice by attaching a sequence to the aptamer strand that is complementary to the linker strand (Scheme 1). Such a plug-and-play methodology enables a highly flexible and modular approach to the sensor platform, in which various binding motifs may be connected to the **M13** chassis *via* DNA-mediated base-paring, without the need to undertake new bioconjugation reactions for different assays.

**SCHEME 2:**
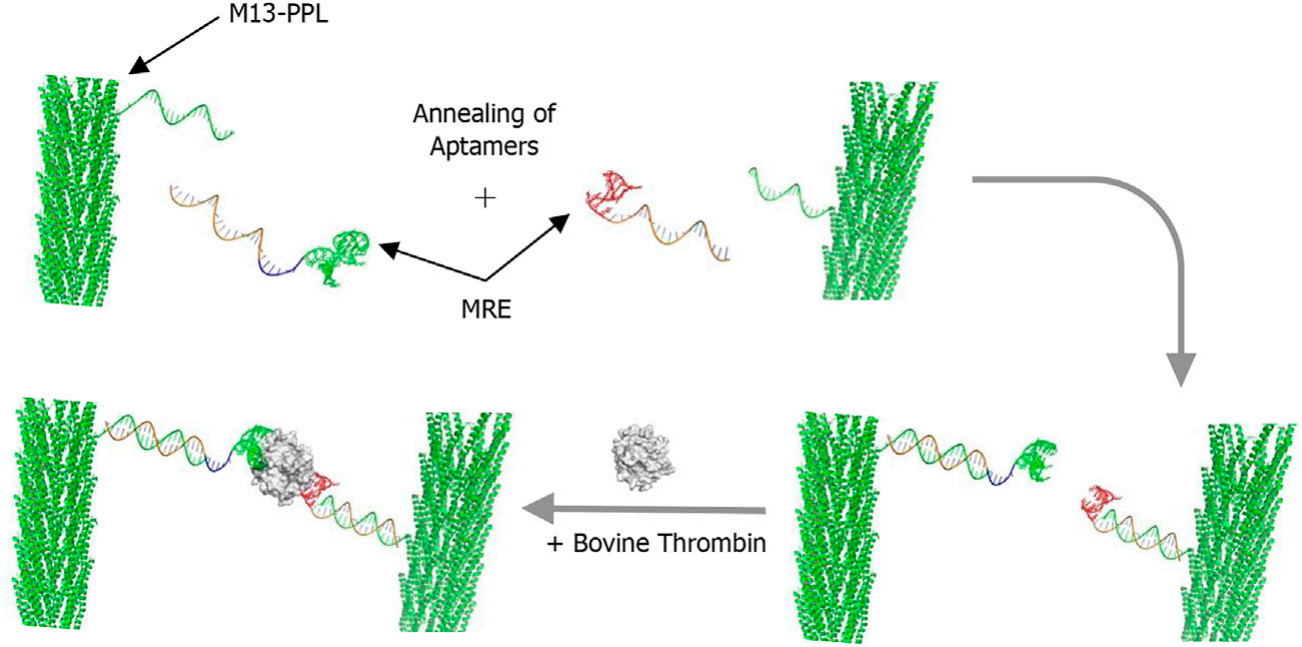
The thrombin sandwich assay design. DNA-modified **M13** bacteriophage (**M13**-**PPL**) is annealed to two different extended aptamers (molecular recognition elements or MREs), to which the target, the protein thrombin, is added for detection. Note: In this study, an average of 15 **PPL** strands were conjugated to each **M13** chassis, although only one is shown here for clarity.

Scheme 2 illustrates the method used for the construction of the molecular components of the thrombin assay system. First the **M13** bacteriophages are prepared in bulk and covalently attached to multiple copies of the universal plug-and-play linker strand **PPL** (here only one strand is shown for clarity) to form the functionalised **M13-PPL** bionanoparticles. Next the two DNA aptamers for thrombin, **TBA** and **HD22**, with extended sequences complementary to **M13**-**PPL**, are annealed *via* DNA duplex formation to complete the assembly of the recognition system. Our hypothesis was that the resulting aggregation of **M13** bionanoparticles brought about by simultaneous sandwich complexation of thrombin by the two aptamers would result in a change in linear alignment in flow and hence a change in the LD signal, as shown schematically in Scheme 1.

### 2.2 Synthesis and characterisation

The sequences of the oligonucleotides used in this study are shown in Table 1. It lists the two aptamers as extended forms (**TBA15** and **HD22T5**), the plug-and-play linker (**PPL**) and a control strand with a scrambled sequence (**NS**). The **PPL** sequence is a 21-mer oligonucleotide containing a thiol moiety at its 3’ end (prepared as a disulfide) and a chromophore tag, 6-carboxyfluorescein (6-FAM), for quantification purposes. This **PPL** strand was conjugated to the bacteriophage via a short linker molecule to form **M13-PPL**, one of four DNA-functionalised phage systems that we reported previously. (Ali et al., 2020). Its preparation, purification using size exclusion chromatography (SEC) and quantification using UV/vis absorbance spectroscopy is outlined in the Supplementary Material S1. A batch of **M13-PPL** from our previous study (Ali et al., 2020) was used again here, which contained an average of fifteen PPL strands conjugated onto its coat protein.

**TABLE 1:** The DNA oligonucleotides used in this study. **PPL**, prepared previously, (Ali et al., 2020) is shown in its thiol form to indicate its attachment point to the **M13** chassis. The two extended aptamers **TBA15** and **HD22T5** are listed with their thrombin binding (aptameric) regions in red and green respectively and their complementary regions to the **PPL** strand in orange.

Name	Sequence
**PPL**	5′-GCCTCACTGATTAAGCATTGG-(6-FAM)-SH-3′
**TBA15**	5′- CCAATGCTTAATCAGTGAGGC GGTTGGTGTGGTTGG -3′
**HD22T5**	5′- CCAATGCTTAATCAGTGAGGC TTTTT AGTCCGTGGTAGGGCAGGTTGGGGTGACT -3′
**NS** (non-specific)	5′-ATG​AGT​ATT​CAA​CAT​TTC​GCC​TCA​CTG​ATT​AAG​CAT​TGG-3′

As described above, the two thrombin aptamers **TBA** and **HD22** can bind at different exosites (secondary binding sites) on the protein surface, making them ideal candidates for a sandwich assay. The interaction of these aptamers with thrombin has been studied in detail since their SELEX screening for **TBA** in 1992 (Bock et al., 1992) and **HD22** in 1997. (Tasset et al., 1997). The G-quadruplex forming **TBA** was extended from its 5’ end with the complementary sequence to that of **PPL** to give the 36-mer **TBA15**. It has previously been shown (De Rache et al., 2012) that elongation of this aptamer sequence does not require a spacer sequence to retain formation of the G-quadruplex. However **HD22** forms a short folded internal duplex in addition to a G-quadruplex. Therefore, to mitigate any adverse effect on this more intricate folded conformation, a short spacer sequence of five thymine bases was inserted between the aptamer sequence and the sequence complementary to **PPL** to give some additional flexibility to the resulting extended 55-mer strand **HD22T5**. CD studies (see Supplementary Material S1) confirmed that sequence elongation of both aptamers had no detrimental effect on their secondary structures.

### 2.3 Binding studies

**FIGURE 1:**
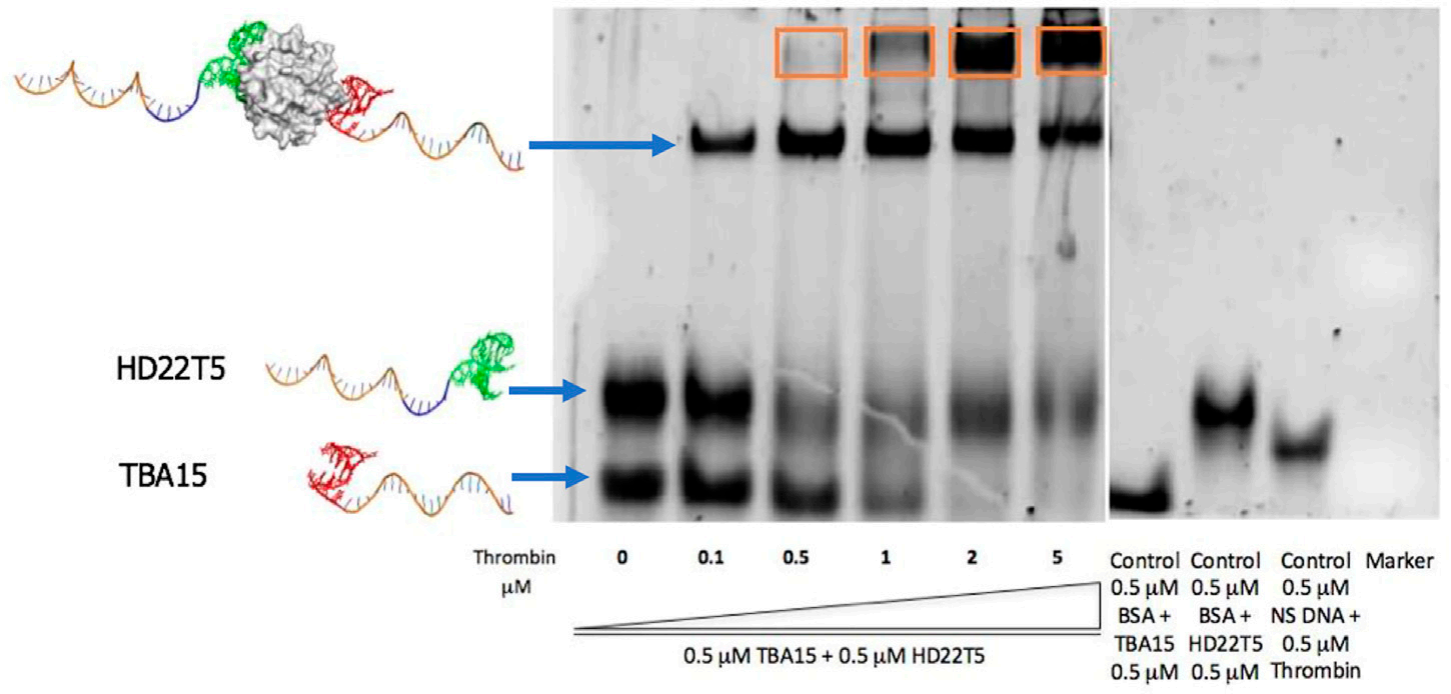
EMSA of **TBA15** and **HD22T5** (both at 0.5 μM) with increasing concentrations of thrombin (0, 0.1, 0.5, 1, 2, 5 μM) (lanes 1–6). Control bovine serum albumin, BSA (0.5 μM) + **TBA15** (0.5 μM), BSA (0.5 μM) + **HD22T5** (0.5 μM), non-specific DNA, **NS** (0.5 μM) + thrombin (0.5 μM) (lanes 7–9). SYBR Gold^®^ nucleic acid stain used to visualize gel. Electrophoresis buffer used: 1.0 x TBE +10 mM KCl. The band in an orange box is ascribed to other stoichiometries or aggregates (see text for details). For more experimental details, see Supplementary Material S1.

Gel electrophoretic mobility shift assays (EMSAs) were undertaken to establish the thrombin binding properties of the extended DNA aptamer strands, with these tested in tandem to establish whether thrombin could be bound by both strands. Accordingly, equimolar amounts of **TBA15** and **HD22T5** were combined and then thrombin was added to each sample in increasing amounts. These mixtures were incubated at 37°C for 30 min to ensure equilibration before each experiment. As shown in an experiment that stained for DNA (Figure 1), upon increasing the concentration of thrombin across the gel (lanes 1–6), **TBA15** became almost completely retained at the top of the gel once a thrombin concentration of 2 µM was reached. Interestingly this change was less dramatic for **HD22T5**, indicating that a higher proportion of this aptamer remained unbound by thrombin at the same concentration. These results can not be explained by differences in binding affinity as the aptamer **HD22** is the stronger binder of thrombin. (Zhao et al., 2021). Instead they are more likely to be ascribed to differences in binding stoichiometry, given that it has previously been shown (Tasset et al., 1997) that two **TBA** aptamers can bind one thrombin molecule simultaneously, whereas this is not the case for **HD22**, which can only bind at exosite II. As for two distinct bands corresponding to thrombin-DNA complexes being observed higher up the gel, it is possible that the lower band, which first appears at sub-stoichiometric concentrations of thrombin, corresponds to the 2/1 sandwich complex, with the higher band (highlighted in an orange box) being the 1/1 complex because of its lower negative charge density. However, an alternative explanation is that different forms or aggregates of thrombin may be bound by these aptamers under these gel conditions, which is supported by other bands for the target being revealed by a protein stain of this gel (see Supplementary Material S1).

**FIGURE 2:**
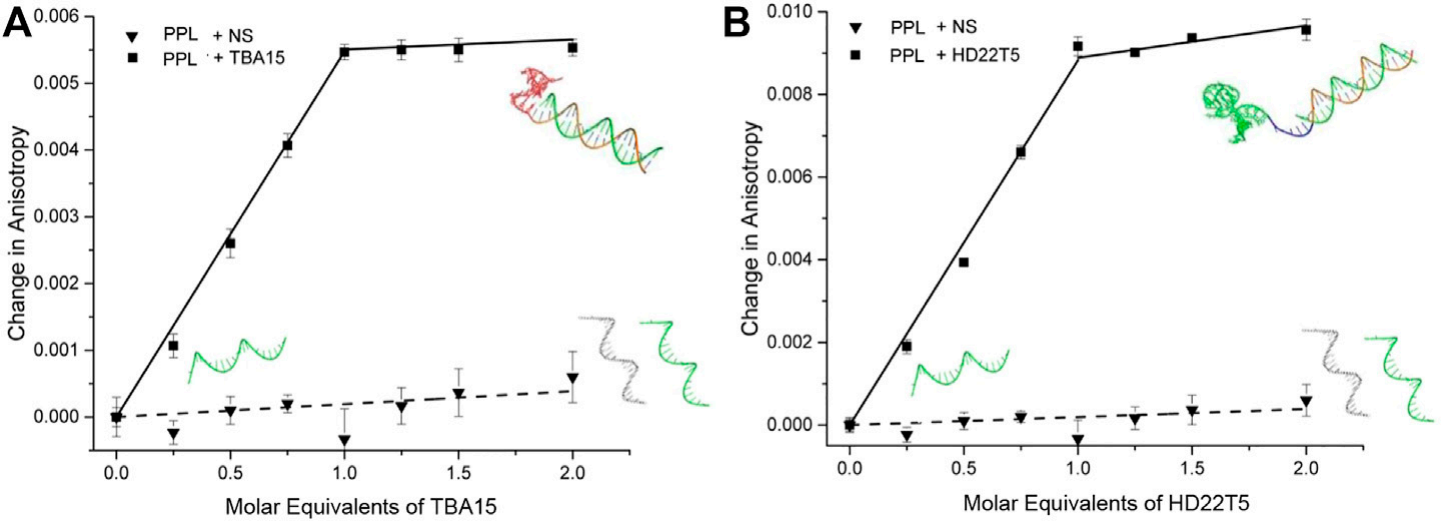
Fluorescence anisotropy changes of **PPL** strand upon the addition of **(A) TBA15** (squares) and **NS** non-specific DNA (triangles), and **(B) HD22T5** (squares) and **NS** non-specific DNA (triangles). [**PPL**] as disulfide **=** 40 nM in the following buffer: 20 mM Tris-HCl, 1 mM MgCl_2_, 120 mM NaCl, 10 mM KCl, 2 mM CaCl_2_, pH 7.4. Measurements performed in triplicate, with error bars presented as a standard error of the mean (SEM).

With the binding of thrombin by both extended aptamers confirmed by EMSAs, their duplex formation in solution with strand **PPL** needed to be confirmed before undertaking the LD sensing studies. Fluorescence anisotropy (FA) was used for this purpose, which involved titrating in **TBA15** and **HD22T5** respectively to solutions of the **PPL** strand. As expected, an increase in the FA signal was observed for both extended aptamers as their molecular tumbling time decreased upon forming a larger complex. (Vafaei et al., 2021). These data indicate that saturation of binding is reached with the addition of one molar equivalent of extended aptamer, corresponding to the formation of a 1/1 duplex in each case (Figure 2). The **HD22T5**-**PPL** duplex had a slightly larger change in FA, which can be explained by **HD22T5** being the larger aptamer. As expected, the addition of a scrambled non-specific strand **NS** (Table 1) brought about no change in FA.

### 2.4 LD sensing studies

With it confirmed that these extended aptamers could bind thrombin as well as form stable duplexes with the individual **PPL** strand, the **PPL**-conjugated M13 bacteriophage bionanoparticles **M13**-**PPL** were then studied using linear dichroism spectroscopy to determine whether the aptamer-mediated detection of thrombin could be achieved. First of all, the extended aptamers were individually incubated with **M13**-**PPL** stocks at physiological pH before being combined at 20.5 nM each to make the **M13**-**PPL**-**TBA15**/**M13**-**PPL**-**HD22T5** sandwich assay sensor platform. The assay solution was then subjected to linear flow in a Couette flow cell, with the LD signal monitored at 225 nm as described previously. (Pacheco-Gómez et al., 2012; Carr-Smith et al., 2015; Ali et al., 2020).

**FIGURE 3:**
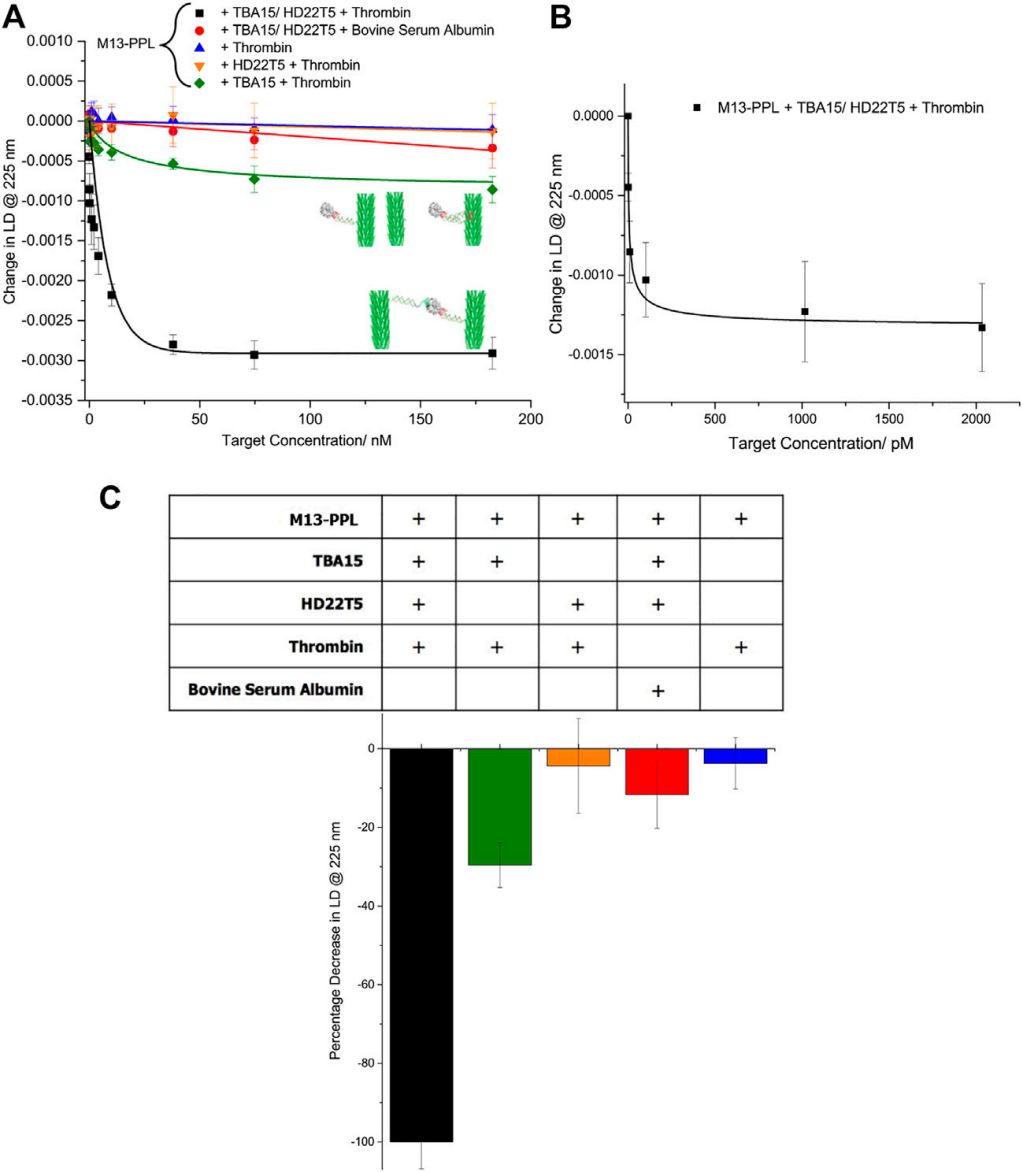
**(A)** Change in LD signal at 225 nm upon addition of targets at increasing concentrations (0–183 nM): **M13**-**PPL**-**TBA15** (20.5 nM) + **M13**-**PPL**-**HD22T5** (20.5 nM) + thrombin (black squares), **M13**-**PPL**-**TBA15** (20.5 nM) + thrombin (green diamonds), **M13**-**PPL**-**HD22T5** (20.5 nM) + thrombin (orange triangles), **M13**-**PPL**-**TBA15** (20.5 nM) + **M13**-**PPL**-**HD22T5** (20.5 nM) + BSE (red circles), **M13**-**PPL** + thrombin; **(B)** Change in LD signal at 225 nm for **M13**-**PPL**-**TBA15** (20.5 nM) + **M13**-**PPL**-**HD22T5** (20.5 nM) + thrombin at increasing concentrations (0, 1.0, 10, 100, 1,000, 2000 p.m.) **(C)** Percentage decrease in LD signal at 225 nm observed at a final target concentration of 183 nM for different conditions used, compared to the full assay system. Experiments performed in the following buffer: 20 mM Tris-HCl, 1 mM MgCl_2_, 120 mM NaCl, 10 mM KCl, 2 mM CaCl_2_, pH 7.4. **M13**-**PPL** was incubated with aptamer (**TBA15** and **HD22T5**) for 30 min before the addition of targets. Measurements performed in triplicate, with error bars presented as a standard error of the mean (SEM). For more details on the LD technique, see Supplementary Material S1.

Upon the addition of thrombin in the picomolar to nanomolar range, a significant decrease in LD signal at 225 nm was observed, resulting from misalignment of the bacteriophage aptamer probes in flow (Figure 3). This result is similar to what was previously observed in the detection of DNA strands, (Ali et al., 2020), with a series of control studies strongly indicating that the sensing system responds well to aggregation of **M13** particles caused by formation of multiple sandwich complexes involving both extended aptamers. These control studies included studies in the presence of one extended aptamer alone, in which only a very small change in signal was observed for **HD22T5**, with a slightly larger change for **TBA15** (an average of 30% of the signal for the combined aptamer set up). This difference is most likely caused by some simultaneous binding by **TBA15** to exosites I and II of thrombin in a 1:2 sandwich complex, as discussed earlier. These results indicate that any 1:1 complex formation with these aptamers does not appear to cause a significant change in LD signal, as this would not trigger **M13** particle aggregation and therefore a change in alignment. Further control experiments with bovine serum albumin (BSA), which is not bound by these aptamers, also showed no significant change in LD signal (red), with an average LD signal decrease of only 8%. Finally, the **PPL**-functionalised **M13** bionanoparticle system was also tested for thrombin in the absence of both aptamers (blue). As expected, no significant change in LD signal was observed (ca. 4% decrease in LD signal), which confirms that no thrombin binding occurs in the absence of the extended aptamers. The data shown in Figure 3B indicates that thrombin can be detected using LD spectroscopy down to the low pM range, which is competitive for this particular target compared to some other more established read-out methods. (Ikebukuro et al., 2004; Xiao et al., 2005; Wang et al., 2008; Cho et al., 2008; Wang and Zhao, 2012; Deng et al., 2014; Kim et al., 2018; Malecka and Ferapontova, 2021; Neupane and Stine, 2021).

## 3 Conclusion

In conclusion, this study reports the first example of an aptamer-functionalised bacteriophage system, enabling the design of a label-free homogenous assay system for non-nucleic acid targets using an LD read-out. The use of LD spectroscopy for sensing offers the potential to test contaminated samples with ease and at speed as no washing or separation steps are required before quantification and the background signal is low. The main advantage of this new plug-and-play LD sensor platform is its modularity in that different aptamer sequences can be connected to a DNA-linked **M13** chassis without performing any new bioconjugation steps to the bacteriophage. If this sandwich assay system were to be taken forward for targets other than thrombin, the same DNA-functionalised chassis could be made as a batch stock of the viral scaffold, with other aptamers functionalised in the same way with sequences complementary to the same **PPL** strand. This would greatly reduce the time taken to develop an assay from aptamer generation to assay testing.

## Data Availability

The raw data supporting the conclusion of this article will be made available by the authors, without undue reservation.

## References

[B1] Alafeef M. Pan D. (2022). Diagnostic approaches for COVID-19: lessons learned and the path forward. ACS Nano 16, 11545–11576. 10.1021/acsnano.2c01697 35921264

[B2] Ali A. Little H. A. Carter J. G. Douglas C. Hicks M. R. Kenyon D. M. (2020). Combining bacteriophage engineering and linear dichroism spectroscopy to produce a DNA hybridisation assay. RSC Chem. Biol. 1, 449–454. 10.1039/d0cb00135j 34458772 PMC8341927

[B3] Bock L. C. Griffin L. C. Latham J. A. Vermaas E. H. Toole J. J. (1992). Selection of single-stranded DNA molecules that bind and inhibit human thrombin. Nature 355, 564–566. 10.1038/355564a0 1741036

[B4] Carr-Smith J. Pacheco-Gómez R. Little H. A. Hicks M. R. Sandhu S. Steinke N. (2015). Polymerase chain reaction on a viral nanoparticle. ACS Synth. Biol. 4, 1316–1325. 10.1021/acssynbio.5b00034 26046486

[B5] Chen C. Liu W. Tian S. Hong T. (2019). Novel surface-enhanced Raman spectroscopy techniques for DNA, protein and drug detection. Sensors 19, 1712. 10.3390/s19071712 30974797 PMC6480126

[B6] Cho H. Baker B. R. Wachsmann-Hogiu S. Pagba C. V. Laurence T. A. Lane S. M. (2008). Aptamer-based SERRS sensor for thrombin detection. Nano Lett. 8, 4386–4390. 10.1021/nl802245w 19367849 PMC3477626

[B7] De Rache A. Kejnovská I. Vorlíčková M. Buess‐Herman C. (2012). Elongated thrombin binding aptamer: A G-quadruplex cation-sensitive conformational switch. Chem. Eur. J. 18, 4392–4400. 10.1002/chem.201103381 22362492

[B8] Deng B. Lin Y. Wang C. Li F. Wang Z. Zhang H. (2014). Aptamer binding assays for proteins: The thrombin example—a review. Anal. Chim. Acta 837, 1–15. 10.1016/j.aca.2014.04.055 25000852

[B9] Ikebukuro K. Kiyohara C. Sode K. (2004). Electrochemical detection of protein using a double aptamer sandwich. Anal. Lett. 37, 2901–2909. 10.1081/al-200035778

[B10] Kim H. An Z. Jang C.-H. (2018). Label-free optical detection of thrombin using a liquid crystal-based aptasensor. Microchem. J. 141, 71–79. 10.1016/j.microc.2018.05.010

[B11] Malecka K. Ferapontova E. E. (2021). Femtomolar detection of thrombin in serum and cerebrospinal fluid via direct electrocatalysis of oxygen reduction by the covalent G4-hemin-aptamer complex. ACS Appl. Mat. Interfaces 13, 37979–37988. 10.1021/acsami.1c03784 33878266

[B12] Michel B. Y. Dziuba D. Benhida R. Demchenko A. P. Burger A. (2020). Probing of nucleic acid structures, dynamics, and interactions with environment-sensitive fluorescent labels. Front. Chem. 8, 112. 10.3389/fchem.2020.00112 32181238 PMC7059644

[B13] Neupane D. Stine K. J. (2021). Electrochemical sandwich assays for biomarkers incorporating aptamers, antibodies and nanomaterials for detection of specific protein biomarkers. Appl. Sci. 11, 7087. 10.3390/app11157087

[B14] Pacheco-Gómez R. Kraemer J. Stokoe S. England H. J. Penn C. W. Stanley E. (2012). Detection of pathogenic bacteria using a homogeneous immunoassay based on shear alignment of virus particles and linear dichroism. Anal. Chem. 84 (1), 91–97. 10.1021/ac201544h 22017566

[B15] Sun Y. Peng Z. Li H. Wang Z. Mu Y. Zhang G. (2019). Suspended CNT-Based FET sensor for ultrasensitive and label-free detection of DNA hybridization. Biosens. Bioelectron. 137, 255–262. 10.1016/j.bios.2019.04.054 31121462

[B16] Tasset D. M. Kubik M. F. Steiner W. (1997). Oligonucleotide inhibitors of human thrombin that bind distinct epitopes. J. Mol. Biol. 272, 688–698. 10.1006/jmbi.1997.1275 9368651

[B17] Trotter M. Borst N. Thewes R. von Stetten F. (2020). Review: Electrochemical DNA sensing – principles, commercial systems, and applications. Biosens. Bioelectron. 154, 112069. 10.1016/j.bios.2020.112069 32056964

[B18] Vafaei S. Allabush F. Tabaei S. R. Male L. Dafforn T. R. Tucker J. H. R. (2021). Förster resonance energy transfer nanoplatform based on recognition-induced fusion/fission of DNA mixed micelles for nucleic acid sensing. ACS Nano 15, 8517–8524. 10.1021/acsnano.1c00156 33961404 PMC8158853

[B19] Wang W. Chen C. Qian M. X. Zhao X. S. (2008). Aptamer biosensor for protein detection based on guanine-quenching. Sens. Actuators, B Chem. 129, 211–217. 10.1016/j.snb.2007.07.125

[B20] Wang X. Zhao Q. (2012). A fluorescent sandwich assay for thrombin using aptamer modified magnetic beads and quantum dots. Microchim. Acta 178, 349–355. 10.1007/s00604-012-0850-1

[B21] Xiao Y. Lubin A. A. Heeger A. J. Plaxco K. W. (2005). Label-free electronic detection of thrombin in blood serum by using an aptamer-based sensor. Angew. Chem. Int. Ed. 44, 5456–5459. 10.1002/anie.200500989 16044476

[B22] Zhao S. Tian R. Wu J. Liu S. Wang Y. Wen M. (2021). A DNA origami-based aptamer nanoarray for potent and reversible anticoagulation in hemodialysis. Nat. Commun. 12, 358. 10.1038/s41467-020-20638-7 33441565 PMC7807036

