# Supplementary material for: A plug-and-play aptamer diagnostic platform based on linear dichroism spectroscopy
Source: Front Chem. 2023 May 9;11:1040873. doi: 10.3389/fchem.2023.1040873 (PMC10203435; doi:10.3389/fchem.2023.1040873)
Supplement: Supplementary file 1 [file DataSheet1.pdf]

Supplementary Information

A plug-and-play aptamer diagnostic platform based on linear dichroism spectroscopy

Haydn A. Little,[1,2] Aysha Ali,[1,2] Jake G. Carter,[1,2] Matthew R. Hicks,[3] Timothy R. Dafforn,[2]* and James H. R. Tucker[1*]

[1] School of Chemistry, University of Birmingham, Edgbaston, Birmingham B15 2TT, UK.

[2] School of Biosciences, University of Birmingham, Edgbaston, Birmingham B15 2TT, UK

[3] Linear Diagnostics Ltd, 97 Vincent Drive, Birmingham B15 2SQ, UK

Contents:

1. DNA strands Page S1

2. M13-Probe synthesis, purification and characterisation Page S2

3. CD Studies Page S4

4. EMSA Gel Studies Page S5

5. LD studies Page S5

1. DNA strands

All DNA strands were bought from commercial suppliers except for **PPL**, which had been prepared and characterized previously.[S1]. The dye 6-carboxyfluorescein dye (6-FAM) and disulfide reagents used for the synthesis of **PPL** are shown below.

PPL sequence (as disulfide): (5'-3'): GCC TCA CTG ATT AAG CAT TGG-(6-FAM)-SSR'

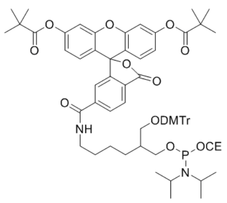

6-FAM

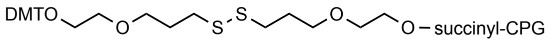

-SS-R'

2. M13-Probe synthesis, purification and characterisation

2.1 Synthesis

The general procedure for **M13** bacteriophage isolation from bacterial culture, the two-step conjugation procedure to DNA, and the subsequent purification and quantification were all performed as described previously.[S1, S2, S3]. A batch of the DNA-functionalised bacteriophage, **M13-PPL** that had been prepared for earlier work[S1] was used again here. For convenience, a summary of the synthetic procedure used is outlined below.

As shown in Scheme S1, the bioconjugation to form **M13-PPL** involved first reacting **M13** with the linker SMCC (succinimidyl 4-(N-maleimidomethyl)cyclohexane-1-carboxylate), which was then coupled with **PPL** as its thiol (stored in tris(2-carboxyethyl)phosphine, TCEP).

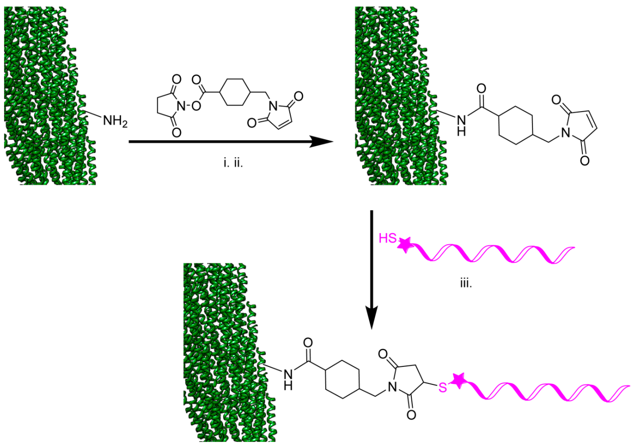

Scheme S1. Conditions to form **M13-PPL**: (i) SMCC in DMSO, 1 h, RT. (ii) glycine, 30 min, RT. (iii) Reaction with the thiol in its reduced thiol form, 16 h, 5 °C. For exact experimental conditions followed for both steps (but using the **PPL** DNA strand in this case), see reference [S2].

2.2 M13-PPL Purification and Characterisation

The conjugated phage **M13-PPL** was purified by SEC (Figure 2a) using an ÄKTA Explorer 10 purification system. The SEC column (Superdex S200 HiLoad 16/60) was first washed with 1.2 column volumes of MilliQ® water and then equilibrated with 1.2 column volumes of 100 mM potassium phosphate buffer, 150 mM NaCl, pH 7.2. The flow rate was set at 1.0 mL/min for 1.2 column volumes with 100 mM potassium phosphate buffer, 150 mM NaCl, pH 7.2. Three absorbances were detected A269, A280 and A495. The eluent collected as 2 mL fractions with a Frac-950 auto collector (Amersham Pharmacia Biotech). Fractions of interest containing the DNA-conjugated phage were combined and concentrated using MWCO spin concentrators by centrifugation at 4000 rpm at 4 °C. Each sample was washed with 1.0 mL of 100 mM potassium phosphate buffer, 150 mM NaCl, pH 7.2 and reduced to 200 µL and the sample removed. The spin concentrator was washed with 3 x 200 µL of 100 mM potassium phosphate buffer, 150 mM NaCl, pH 7.2 and added to the sample. As described previously[S1, S2], the average number of DNA strands appended to each chassis in M13-PPL was estimated as 15 from the UV/vis spectrum (Figure 2b) by knowing the molar extinction coefficients of the M13 phage at 269 nm and 6-FAM at 495 nm.

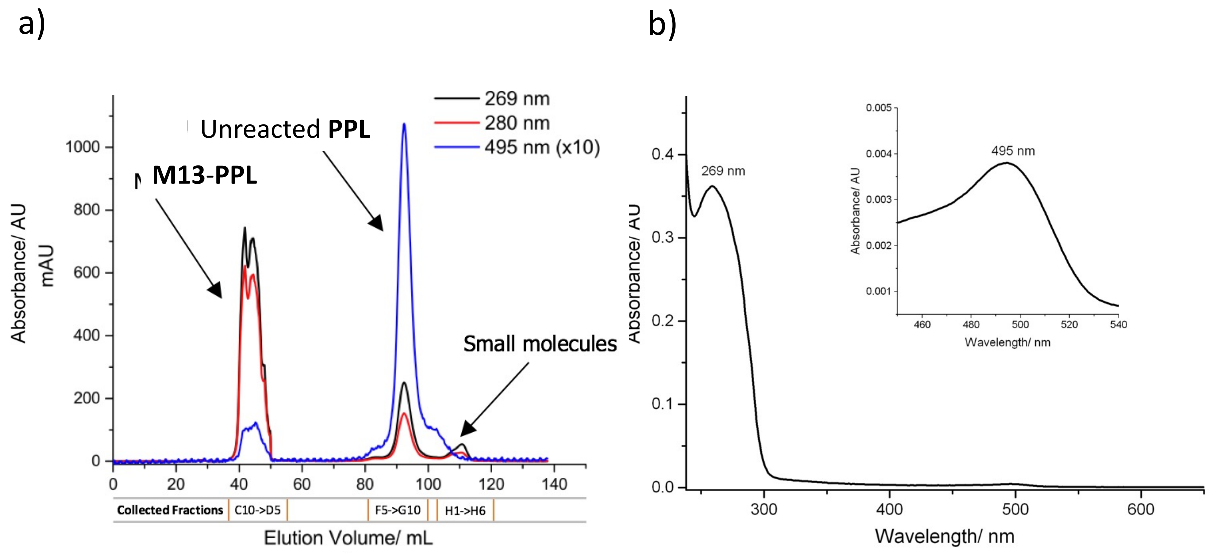

Figure S2. a) Size exclusion chromatogram (SEC) of **M13-PPL** conjugates, unreacted **PPL** and small molecules. Multiple absorbance wavelengths monitored: 269 nm (black) for M13 bacteriophage, 280 nm (red) proteins, 495 nm (blue) 6-FAM tag on **PPL**; b) UV/vis spectrum of **M13-PPL** showing the 6-FAM absorption at 495 nm (100 mM potassium phosphate buffer, 150 nM NaCl, at pH 7.2, at RT).

3. CD studies

Circular dichroism (CD) experiments were carried out using a Jasco J-1500 spectropolarimeter (Jasco, Japan) at 293 K using a 1 cm pathlength 6Q quartz cuvette. A baseline was recorded using the corresponding buffer to the sample and subtracted from the sample spectra. All measurements were conducted with the following parameters: Range 190–350 nm, Response 1s, Data pitch 0.5 nm, Scanning mode Continuous, Scan speed 50 nm/min, Band width 1 nm, and Accumulation 3. The CD spectra (Fig. S1) of the original (**TBA** and **HD22**) and extended (**TBA15** and **HD22T5**) aptamers were very similar in three different solvent conditions (water alone, water with KCl added to induce folding and thrombin binding (TB) buffer).

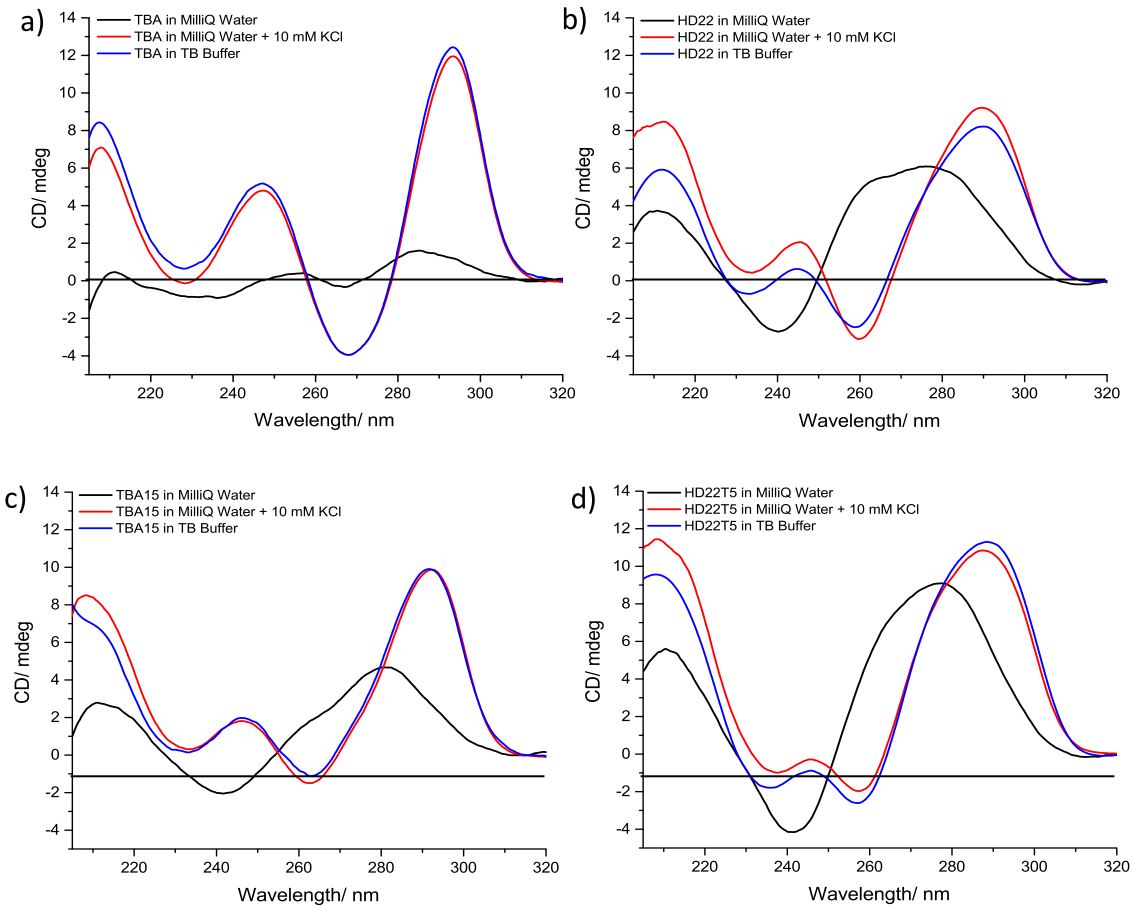

Figure S1. CD spectra of the two thrombin binding aptamers and their respective modifications: a) unextended aptamer **TBA**, b) unextended aptamer **HD22**, c) **TBA15**, d) **HD22T5**. Experiments performed at 2 µM in Milli-Q® water (black), with 10 mM KCl added (red) and also in thrombin binding (TB) buffer as follows: 20 mM Tris-HCl, 1 mM $MgCl_2$, 120 mM NaCl, 10 mM KCl, 2 mM $CaCl_2$, pH 7.4 (blue).

4. EMSA Gel Studies

Each sample was prepared in 20 mM Tris-HCl, 1 mM $MgCl_2$, 120 mM NaCl, 10 mM KCl, 2 mM $CaCl_2$, pH 7.4 and 3% glycerol. All aptamer strands were denatured at 90 °C for 5 minutes and allowed to cool at RT slowly before addition to thrombin or BSA. These mixtures were incubated at 37 °C for 30 minutes to ensure equilibration. Gel EMSAs were performed using 12% acrylamide 0.8% bisacrylamide with 10 mM potassium chloride, 1 x TBE, 10% APS (140 µL) and TEMED (70 µL) in a 1 x TBE + 10 mM potassium chloride, pH 8.3 electrophoresis buffer. The gels were performed at 100 V for 120 min and visualized with SYBR GOLD® nucleic acid stain (15 min) using an ultraviolet transilluminator fitted with a camera. Post DNA staining was conducted with InstantBlue™Ultrafast Protein Stain for 15 minutes, with subsequent treatment with water (15 min). A protein stain of the same gel used for DNA visualisation (main text Figure 1) indicated a series of protein bands for the thrombin sample alone (Figure S2).

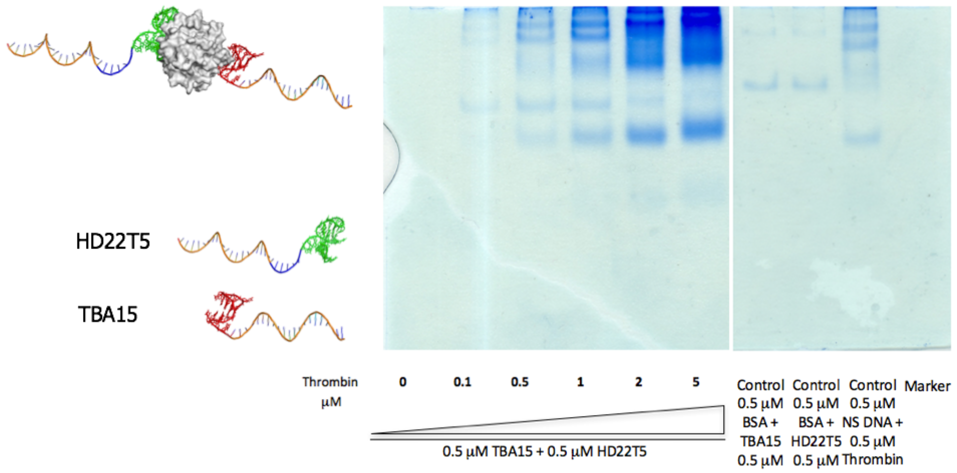

Figure S2. EMSA of **TBA15** and **HD22** (both at 0.5 µM) with increasing concentrations of thrombin (0, 0.1, 0.5, 1, 2, 5 µM) (lanes 1-6). Control bovine serum albumin, BSA (0.5 µM) + **TBA15** (0.5 µM), BSA (0.5 µM) + **HD22T5** (0.5 µM), non-specific DNA, **NS** (0.5 µM) + thrombin (0.5 µM) (lanes 7-9). InstantBlue™ Ultrafast Protein Stain used to visualize gel. Electrophoresis buffer used: 1.0 x TBE + 10 mM KCl.

5. LD Studies

LD spectroscopy was carried out according to previously reported procedures [S1, S2, S3]. For this this work, a Jasco J-715 spectropolarimeter (Jasco, Japan) was used, which had been modified for linear dichroism spectroscopy. Samples (60 µL) were placed into a micro-couette cell apparatus within the LD sample chamber. A baseline was recorded (with a non-rotating capillary) and subtracted from the sample with a couette rotating at 3 V and the signal zeroed at 800 nm. All measurements were conducted with the following parameters: Range 190–800 nm, Sensitivity 0.1 dOD, Data pitch 1.0 nm, Scanning mode continuous, Scan speed 200 nm/min, Band width 2 nm, and Accumulation 3. Initial samples were incubated for 30 min before addition of target aliquots, with an additional incubation time of 10 minutes after each addition. The LD spectrum of **M13-PPL** was very similar to that of unfunctionalised **M13**, as reported previously[S1].

References:

[S1] A. Ali, H. A. Little, J. G. Carter, C. Douglas, M. R. Hicks, D. M. Kenyon, C. Lacomme, R. T. Logan, T. R. Dafforn, J. H. R. Tucker, *RSC Chem. Biol.*, 2020, 1, 449-454.

[S2] J. Carr-Smith, R. Pacheco-Gómez, H. A Little, M. R Hicks, S. Sandhu, N. Steinke, D. J Smith, A. Rodger, S. A Goodchild, R. A Lukaszewski, J. H. R. Tucker, T. R. Dafforn, *ACS Synth. Biol.*, 2015, 4, 1316-25.

[S3] H. A. Little, *The development of novel diagnostic sensors based on linear dichroism spectroscopy*, 2017, University of Birmingham. Ph.D. https://etheses.bham.ac.uk//id/eprint/7421/
